# Human PRV Infection in China: An Alarm to Accelerate Eradication of PRV in Domestic Pigs

**DOI:** 10.1007/s12250-021-00347-1

**Published:** 2021-02-04

**Authors:** Zhenhua Guo, Xin-Xin Chen, Gaiping Zhang

**Affiliations:** 1 Key Laboratory of Animal Immunology of the Ministry of Agriculture, Henan Provincial Key Laboratory of Animal Immunology, Henan Academy of Agricultural Sciences, Zhengzhou, 450002 China; 2 College of Veterinary Medicine, Henan Agricultural University, Zhengzhou, 450002 China

Pseudorabies, also known as Aujeszky’s disease, is one of the most economically important viral diseases in pigs and is lethal to other susceptible animals (Ren *et al*. 2020). The causative agent, pseudorabies virus (PRV), is an enveloped virus with a large double-stranded DNA genome encoding at least 70 proteins (Wong *et al*. 2019). PRV belongs to the family *Herpesviridae*, subfamily *Alphaherpesvirinae*, genus *Varicellovirus* and infects multiple animals, such as pigs, dogs, cats, rabbits, cattle, sheep, goats, minks, foxes, wolves, lynxes, etc. (He *et al*. 2019; Laval and Enquist 2020). Pigs are recognized as the natural hosts for the virus, and the PRV infection causes severe neurological symptoms in piglets with almost 100% mortality, respiratory/neurological signs in the nursery, respiratory signs in adult pigs, and reproductive disorders in sows (He *et al*. 2019; Ren *et al*. 2020). Most of the other infected non-natural animal hosts die within 24–48 h of disease onset, which is usually characterized by severe pruritus in the head and neck, accompanied by self-mutilation (Laval and Enquist 2020).

Although cases of suspected PRV infections in humans have been occasionally reported in Europe, the occurrence of PRV infection in humans is still controversial owing to the lack of a definitely etiological or serological diagnosis (Wong *et al.* 2019). However, since 2018, several reports from China have shown that PRV could infect humans through detection of PRV nucleic acids by metagenomic next-generation sequencing (NGS) or specific antibody for PRV by ELISA (Fig. 1A, Table 1) (Zhao *et al.* 2018; Ai *et al*. 2018; Fan *et al*. 2020; Hu *et al*. 2020; Li *et al.* 2020; Wang *et al*. 2019, 2020; Yang H *et al.* 2019; Yang X *et al.* 2019; Zheng *et al*. 2019). Especially, Liu *et al.* successfully isolated a PRV strain (hSD-1/2019, GenBank no. MT468550) from a patient with acute encephalitis, providing direct evidence of PRV infection in humans (Liu *et al*. 2020). All 23 patients in the above study were in close contact with pigs or pork, and consisted of butchers, pork dealers, cooks, veterinarians, and swineherds (Fig. 1, Table 1). Most of these patients were either injured at work or their eyes were directly exposed to pollutants. Human-to-human transmission was not found. At the early stage of infection (usually within 7 days), a “flu-like” symptoms were observed, including fever (100%, 23/23), respiratory signs (72.7%, 16/22), and headache (57.9%, 11/19). Then, these symptoms rapidly progressed to neurological disorders after disease onset, including seizures/convulsions (95.7%, 22/23) and disturbance of consciousness (95.7%, 22/23). In addition, 60% (12/20) of the patients showed severe visual impairment and 77.3% (17/22) complicated by pulmonary inflammation. All patients were diagnosed with viral encephalitis, except for one patient who was diagnosed with endophthalmitis alone, probably because her eyes were directly exposed to sewage on a pig farm. Despite receiving systematic antiviral treatment, the patients had a very poor prognosis; 17.4% (4/23) of the patients died, 17.4% (4/23) developed blindness, and 21.7% (5/23) patients experienced severe visual impairment. In fact, at the time of writing this study, 65.2% (15/23) of the patients still had severe central nervous system symptoms such as persistent vegetative status, and memory loss, and only could follow simple instructions. A recent retrospective seroepidemiologic survey using 1335 serum samples collected from patients with encephalitis from China between 2012 and 2017 revealed that 6.52%–14.25% of the patients had specific PRV-gB antibodies (Li *et al*. 2020). All these evidences suggest that PRV is an emerging zoonotic pathogen that can infect humans in particular conditions, although the risk of infection remains low. Therefore, PRV should be included in the differential diagnosis of patients with viral encephalitis, especially those who are in close contact with pigs or pork.

**Fig. 1:**
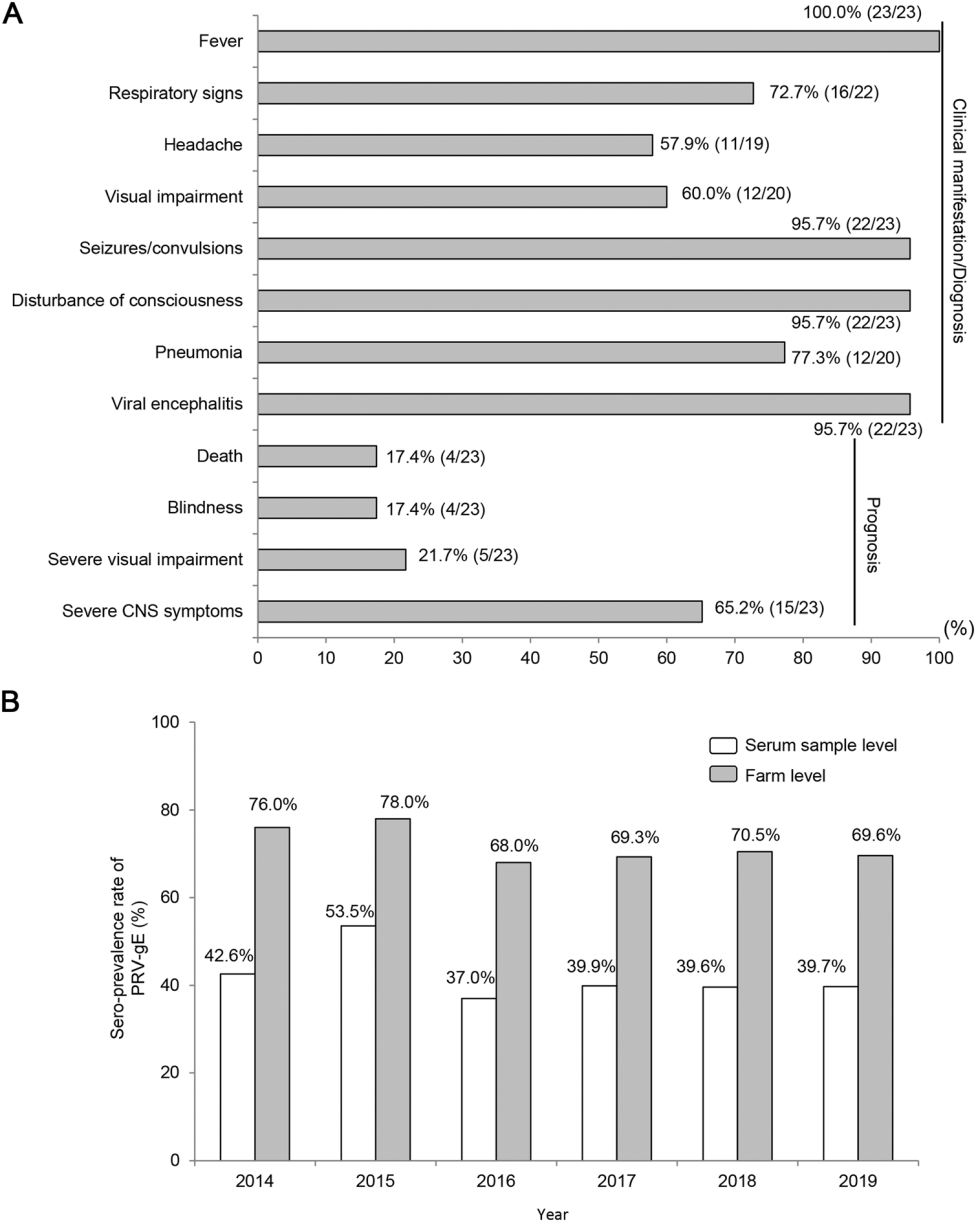
Clinical characteristics of PRV infection patients and sero-prevalence of PRV-gE in domestic pigs in China. **A** Clinical manifestations and prognosis of PRV infection patients in China. At the early stage of infection, fever (100%, 23/23), respiratory signs (72.7%, 16/22) and headache (57.9%, 11/19) were often observed. Then, rapidly progressed to neurological disorders, including seizures/convulsions (95.7%, 22/23) and disturbance of consciousness (95.7%, 22/23). Additionally, 60% (12/20) of the patients had severe visual impairment and 77.3% (17/22) complicated by pulmonary inflammation. All patients were diagnosed with viral encephalitis, except for one who was diagnosed with endophthalmitis alone. The prognosis was very bad: 17.4% (4/23) of the patients died, 17.4% (4/23) developed blindness, 21.7% (5/23) showed severe visual impairment, and 65.2% (15/23) of the patients had severe central nervous system (CNS) symptoms such as persistent vegetative status, memory loss and only could follow simple instructions. **B** Sero-prevalence of PRV-gE in central China from 2014 to 2019. The positive rate of PRV-gE antibodies ranged from 37.0 to 53.5% and from 68.0 to 78.0% at serum sample level and pig farm level, respectively.

**Table 1:** Summary information of the human PRV infection cases in China.

Case	Common information						Clinical manifestation								
	Age (y)	Sex	Occupation	Contact history	Place of infection	Year	Fever	Respiratory failure	Headache	Pneumonia	Visual impairment	Seizures	Disturbance of consciousness	Viral encephalitis	Ocular inflammation
1	38	Male	Butcher	Injury	Shandong	2017	Yes	Yes	Yes	Yes	Yes	Yes	Yes	Yes	N.A
2	55	Male	Pork dealer	Pork	Inner Mongolia	2017	Yes	Yes	No	Yes	Yes	Yes	Yes	Yes	N.A
3	51	Male	Cook	Pork	Inner Mongolia	2017	Yes	Yes	Yes	Yes	No	Yes	Yes	Yes	N.A
4	42	Female	Butcher	Pigs/pork	Hebei	2016	Yes	No	No	No	Yes	Yes	Yes	Yes	Yes
5	46	Female	Swineherd	Sewage	Jiangxi	2017	Yes	No	Yes	No	Yes	No	No	No	Yes
6	44	Male	Sick pig handler	Diseased pigs; pork	Shandong	2019	Yes	Yes	No	Yes	Yes	Yes	Yes	Yes	Yes
7	59	Male	Swineherd	Needle injury	Hebei	2019	Yes	No	No	No	Yes	Yes	Yes	Yes	N.A
8	50	Male	Butcher	Injury at work	Shandong	2018	Yes	No	Yes	Yes	No	Yes	Yes	Yes	N.A
9	50	Female	Pork cutter	Injury at work	Shandong	2018	Yes	Yes	Yes	Yes	No	Yes	Yes	Yes	N.A
10	43	Male	Sick pig handler	Injury at work	Shandong	2018	Yes	Yes	No	Yes	Yes	Yes	Yes	Yes	N.A
11	59	Male	Pork cutter	Injury at work	Shandong	2018	Yes	Yes	No	Yes	No	Yes	Yes	Yes	N.A
12	50	Male	Pork cutter	N.A	Shandong	2018	Yes	Yes	No	Yes	Yes	Yes	Yes	Yes	N.A
13	46	Male	Veterinarian	Injury/ dead pigs	Shandong	2018	Yes	No	Yes	No	No	Yes	Yes	Yes	N.A
14	25	Male	Veterinarian	Diseased pigs	Hubei	2018	Yes	No	Yes	Yes	Yes	Yes	Yes	Yes	N.A
15	25	Male	Butcher	Pigs/pork	Henan	2019	Yes	Yes	Yes	Yes	No	Yes	Yes	Yes	N.A
16	49	Male	Butcher	Pork	Henan	2019	Yes	Yes	Yes	Yes	No	Yes	Yes	Yes	N.A
17	43	Male	Veterinarian	Diseased pigs	Shandong	2018	Yes	Yes	Yes	Yes	No	Yes	Yes	Yes	N.A
18	44	Male	Pork vendor	Injury	Anhui	2019	Yes	Yes	No	No	Yes	Yes	Yes	Yes	N.A
19	N.A	Male	Butcher	N.A	Shandong	2018	Yes	Yes	N.A	Yes	N.A	Yes	Yes	Yes	N.A
20	N.A	Male	Swineherd	N.A	Hebei	2018	Yes	Yes	N.A	Yes	Yes	Yes	Yes	Yes	N.A
21	N.A	Male	Driver	N.A	Guangdong	2018	Yes	Yes	N.A	Yes	N.A	Yes	Yes	Yes	N.A
22	N.A	Female	Pork dealer	N.A	Beijing	2011	Yes	Yes	N.A	Yes	N.A	Yes	Yes	Yes	N.A
23	49	Male	Slaughterer	N.A	Anhui	2019	Yes	N.A	Yes	N.A	Yes	Yes	Yes	Yes	Yes

Case	Diagnosis			Clinical outcome	References
	Vitreous humor (NGS)	Cerebrospinal fluid (NGS)	Antibody (ELISA)		
1	N.A	+	+	Minimally conscious state	Zhao *et al*. (2018)
2	N.A	+	+	Death	
3	N.A	+	+	Death	
4	N.A	N.A	N.A	Minimally conscious state; Blindness	
5	+	–	+	Visually impaired	Ai *et al*. (2018)
6	+	+	N.A	Persistent vegetative state; Visually impaired	Wang *et al*. (2019)
7	N.A	+	+	Follow simple instructions; Mild cognitive deficit; Blindness	Zheng* et al*. (2019)
8	N.A	+	N.A	Slow responses, Occasional seizures	Yang X *et al*. (2019)
9	N.A	+	N.A	Ventilator-dependent	
10	N.A	+	N.A	Follow simple instructions; Blurry vision	
11	N.A	+	N.A	Ventilator-dependent	
12	N.A	+	N.A	Slow responses; Blindness	
13	N.A	+	+	N.A	Yang H *et al*. (2019)
14	–	+	+	Blindness	Liu *et al.* (2020)
15	–	+	+	Mild memory impairment	
16	–	+	+	Minimally conscious state	
17	–	+	+	Persistent vegetative state	
18	N.A	+	+	Follow simple instructions; blurry vision	Wang *et al*. (2020)
19	N.A	+	+	Death	Fan *et al.* (2020)
20	N.A	+	+	Memory loss and bilateral hand tremor; Visual impairment	
21	N.A	+	+	Follow simple instructions	
22	N.A	+	–	Death	
23	+	N.A	N.A	N.A	Hu *et al*. (2020)

*N.A*. not available.

Genetically, PRVs are divided into Genotype 1 and Genotype 2 (He *et al.* 2019). Genotype 1 (Kaplan, GenBank no. JF797218) contains several subgroups and is mainly distributed in Europe and North America and have been eradicated in domestic pigs in most of these regions, whereas Genotype 2 is prevalent in China and is further classified into two sub-genotypes: Genotype 2.1 (Ea, GenBank no. KU315430), also known as classical PRVs, which were more common before 2011 and Genotype 2.2 (HeN1, GenBank no. KP098534), also known as novel PRV variants, which were first observed in 2011 and have been dominant since then (He *et al*. 2019). The average amino acid difference between Genotype 2.2 and Genotype 2.1 was about 1.16%. However, with Genotype 1 strains this value becomes 4.94%. And multiple amino acids difference also has been found among the 69 protein coding regions (He *et al*. 2019; Zhai *et al*. 2019). The emergence of PRV variants (Genotype 2.2) drastically reduced the efficacy of the available commercial vaccines (Ren *et al*. 2020). Seroepidemiologic surveillance conducted by our lab since 2014 showed that about 40.0% of serum samples and 70.0% of pig farms were positive for PRV-gE in Henan Province, central China (Fig. 1B, Table 2). Thus, PRVs severely affect the pig industry in China and pose a new threat to individuals who are in close contact with pigs or pork. However, limited genetic information regarding PRV strains derived from human cases is available. Only 1 of the 23 cases in the previously mentioned study provided the genomic information of the PRV strain (hSD2019) that was identified as a novel PRV variant, which was belonging to Genotype 2.2 (Liu *et al*. 2020). The limited information dampens our knowledge to recognize the genetic characteristics of human PRV strains and understand the co-opting evolution of PRV cross-species. Whether there exist differences in the pathogenicity of classical and variant PRVs in humans and the underlying molecular mechanisms remain unclear. And whether the existing attenuated vaccines are safe enough for humans is also unknown.

**Table 2:** Sero-prevalence of PRV-gE in central China from 2014 to 2019.

Year	Sample level			Farm level		
	Sample numbers	gE-Positive numbers	Sero-prevalence rate (%)	Farm numbers	gE-Positive numbers	Sero-prevalence rate (%)
2014	8244	3509	42.6	436	331	76.0
2015	5304	2838	53.5	316	246	78.0
2016	7179	2654	37.0	381	259	68.0
2017	7028	2801	39.9	342	237	69.3
2018	4052	1605	39.6	264	186	70.5
2019	1107	439	39.7	79	55	69.6

Another concern is whether there is a correlation between PRV human cases and the PRV infection level in pigs? Actually, it is not easy to answer this question. Historically, there were only rare reports of human PRV infection cases abroad (about six reports before 1992) (Wong *et al*. 2019). In China, limited by the development of diagnostic technology (NGS) and clinical medical cognition, PRV infection screening was not included in most of the encephalitis cases before 2010. Thus, we lack the early data on human PRV infections in our country. Since 2011, the emergence of novel PRV variants has led to the high prevalence of PRV in domestic pigs. And some human cases of PRV infection have been reported in China since 2018, but it is still an extremely accidental infection relative to the large number of researchers and workers in the breeding and slaughtering industry. And most of the PRV cases were diagnosed through detection of PRV nucleic acids by NGS or specific antibody for PRV by ELISA, which lack the genetic information of PRV strains derived from human cases. Therefore, it is difficult to say there is a correlation between infected people and the high prevalence of PRV in pigs according to available data. However, accelerating PRV eradication in pigs does help eliminate this potential risk factor and effectively slow the cross-species evolution of PRV.

Currently, our knowledge regarding the pathogenesis and host immunity of the encephalitis caused by PRV infection is still limited, and no guideline for the treatment of PRV exists. In this regard, some key points should be considered. As the current vaccines do not prevent infections caused by the novel PRV variants, the development of more safe and effective vaccines based on the PRV variants is warranted to reduce PRV transmission in pigs in China. Boost detection of PRV in viral encephalitis cases in order to obtain more genetic characteristics of human PRVs would help clarify the mechanisms of PRV cross-species. In addition, the development of targeted drugs and treatment options is important to improve the efficacy and prognosis of infected patients, as PRV-caused encephalitis has a very poor prognosis and current treatments fail to prevent the progress of the disease. Moreover, researchers and workers in the breeding and slaughtering industry should increase their knowledge and awareness regarding self-protection, especially in PRV polluted working environment, and ensure that they always wear gloves, prevent trauma, wash hands frequently, and do not rub their eyes with dirty hands. More importantly, in the long run, it is imperative to actively promote the eradication of PRV in domestic pigs, which will greatly slow down the adaptive evolution of PRV cross-species, thereby preventing the imminent risk to individuals engaged in occupations that involve close contact with the animals.

## References

[CR1] Ai J, Weng S, Cheng Q, Cui P, Li Y, Wu H, Zhu Y, Xu B, Zhang W (2018). Human endophthalmitis caused by pseudorabies virus infection, China, 2017. Emerg Infect Dis.

[CR2] Fan S, Yuan H, Liu L, Li H, Wang S, Zhao W, Wu Y, Wang P, Hu Y, Han J, Lyu Y, Zhang W, Chen P, Wu H, Gong Y, Ma Z, Li Y, Yu J, Qiao X, Li G, Zhao Y, Wang D, Ren H, Peng B, Cui L, Wang J, Guan H (2020). Pseudorabies virus encephalitis in humans: a case series study. J Neurovirol.

[CR3] He W, Auclert LZ, Zhai X, Wong G, Zhang C, Zhu H, Xing G, Wang S, He W, Li K, Wang L, Han GZ, Veit M, Zhou J, Su S (2019). Interspecies transmission, genetic diversity, and evolutionary dynamics of pseudorabies virus. J Infect Dis.

[CR4] Hu F, Wang J, Peng X (2020). Bilateral necrotizing retinitis following encephalitis caused by the pseudorabies virus confirmed by next-generation sequencing. Ocul Immunol Inflamm.

[CR5] Laval K, Enquist LW (2020). The neuropathic itch caused by pseudorabies virus. Pathogens.

[CR6] Li XD, Fu SH, Chen LY, Li F, Deng JH, Lu XC, Wang HY, Tian KG (2020). Detection of pseudorabies virus antibodies in human encephalitis cases. Biomed Environ Sci.

[CR7] Liu Q, Wang X, Xie C, Ding S, Yang H, Guo S, Li J, Qin L, Ban F, Wang D, Wang C, Feng L, Ma H, Wu B, Zhang L, Dong C, Xing L, Zhang J, Chen H, Yan R, Wang X, Li W (2020). A novel human acute encephalitis caused by pseudorabies virus variant strain. Clin Infect Dis.

[CR8] Ren J, Wang H, Zhou L, Ge X, Guo X, Han J, Yang H (2020). Glycoproteins C and D of PRV Strain HB1201 contribute individually to the escape from Bartha-K61 vaccine-induced immunity. Front Microbiol.

[CR10] Wang Y, Nian H, Li Z, Wang W, Wang X, Cui Y (2019). Human encephalitis complicated with bilateral acute retinal necrosis associated with pseudorabies virus infection: a case report. Int J Infect Dis.

[CR9] Wang D, Tao X, Fei M, Chen J, Guo W, Li P, Wang J (2020). Human encephalitis caused by pseudorabies virus infection: a case report. J Neurovirol.

[CR11] Wong G, Lu J, Zhang W, Gao GF (2019). Pseudorabies virus: a neglected zoonotic pathogen in humans?. Emerg Microbes Infect.

[CR12] Yang H, Han H, Wang H, Cui Y, Liu H, Ding S (2019). A case of human viral encephalitis caused by pseudorabies virus infection in China. Front Neurol.

[CR13] Yang X, Guan H, Li C, Li Y, Wang S, Zhao X, Zhao Y, Liu Y (2019). Characteristics of human encephalitis caused by pseudorabies virus: a case series study. Int J Infect Dis.

[CR14] Zhai X, Zhao W, Li K, Zhang C, Wang C, Su S, Zhou J, Lei J, Xing G, Sun H, Shi Z, Gu J (2019). Genome characteristics and evolution of pseudorabies virus strains in Eastern China from 2017 to 2019. Virol Sin.

[CR15] Zhao W, Wu Y, Li H, Li S, Fan S, Wu H, Li Y, Lu Y, Han J, Zhang W, Zhao Y, Li G, Qiao X, Ren H, Zhu Y, Peng B, Cui L, Guan H (2018). Clinical experience and next-generation sequencing analysis of encephalitis caused by pseudorabies virus. Zhonghua Yi Xue Za Zhi.

[CR16] Zheng L, Liu X, Yuan D, Li R, Lu J, Li X, Tian K, Dai E (2019). Dynamic cerebrospinal fluid analyses of severe pseudorabies encephalitis. Transbound Emerg Dis.

